# Endothelial cells and coagulation

**DOI:** 10.1007/s00441-021-03471-2

**Published:** 2021-05-20

**Authors:** Katharina Neubauer, Barbara Zieger

**Affiliations:** Department of Pediatrics and Adolescent Medicine, Division of Pediatric Hematology and Oncology, Medical Center, Faculty of Medicine, University of Freiburg, Freiburg, Germany

**Keywords:** Hemostasis, Thrombosis, Platelets, Von Willebrand factor, Endothelial injury

## Abstract

Endothelial cells form a monolayer, which lines blood vessels. They are crucially involved in maintaining blood fluidity and providing controlled vascular hemostasis at sites of injury. Thereby endothelial cells facilitate multiple mechanisms, including both procoagulant and anticoagulant, which must be kept in balance. Under physiological conditions, endothelial cells constitute a nonadhesive surface preventing activation of platelets and the coagulation cascade. Multiple fibrinolytic and antithrombotic properties act on their cell surface contributing to the maintenance of blood fluidity. These include platelet inhibition, the heparin-antithrombin III system, tissue factor pathway inhibition, thrombomodulin/protein C system, and fibrinolytic qualities. At sites of vascular damage, platelets react immediately by adhering to the exposed extracellular matrix, followed by platelet-platelet interactions to form a clot that effectively seals the injured vessel wall to prevent excessive blood loss. For solid thrombus formation, functional platelets are essential. In this process, endothelial cells serve as a support surface for formation of procoagulant complexes and clotting. This review gives an overview about the central role of the endothelium as a dynamic lining which controls the complex interplay of the coagulation system with the surrounding cells.

## Background

The endothelium is a monolayer of cobblestone-shaped cells, which covers the inner wall of blood vessels separating the lumen from the surrounding tissue as a cellophane-like barrier. Although the endothelium is < 0.2 µm thick, it weights approximately 1 kg in an average-sized human and covers a total surface area of 4000 to 7000 m^2^ (Wolinsky 1980). Endothelial cells (ECs) are central and active parts of two major systems in the body—the immune and the vascular system. Depending on the tissue of origin, structure of the ECs varies (Gomez-Salinero and Rafii 2018). The functions of ECs are versatile and include regulating transport from the blood to underlying cells and tissues, permeability, vascular tone, cellular adhesion, smooth muscle cell proliferation, angiogenesis, and vessel wall inflammation. ECs are able to respond to physical and chemical signals by production of a wide range of factors that regulate these processes.

This review focuses on specific functions and adaptations of ECs maintaining blood fluidity as well as preventing thrombus formation and extravascular blood loss. This is only possible because ECs actively regulate the blood coagulation system and the production of solutes, hormones, or macromolecules (Mehta and Malik 2006).

## Endothelial cell function

### Platelet inhibitory properties of the endothelium

**Fig. 1:**
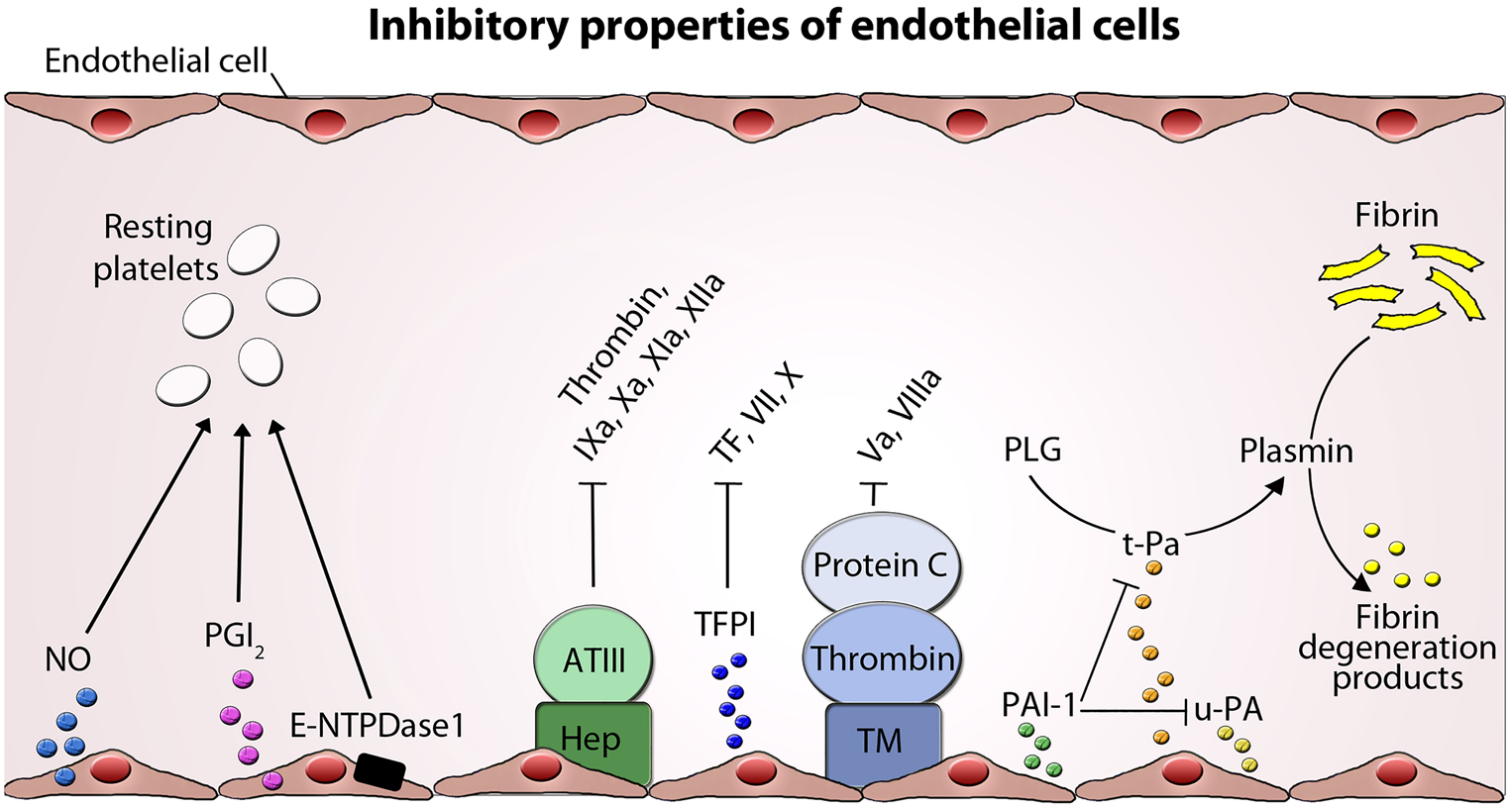
Inhibitory properties of endothelial cells. Inhibition of platelet function and coagulation by several endothelial molecules and factors and the targeted coagulation factors are shown. Endothelial cells (ECs) express nitric oxide (NO), prostacyclin (PGI_2_), and ectonucleoside triphosphate diphosphohydrolase-1 (E-NTPDase1), which inhibit platelet adhesion and aggregation. Heparin-like molekules (Hep) expressed on endothelial surface serve as a cofactor for antithrombin III (ATIII) inactivating several coagulation factors. ECs express tissue factor pathway inhibitor (TFPI), which limits the action of tissue factor (TF) and inhibits excessive TF-mediated activation of coagulation factors VII and X. Thrombomodulin (TM) binds thrombin activating protein C and degrades factor Va and VIIIa. Activation of fibrinolysis by endothelial tissue plasminogen activator (t-PA) and urokinase-type plasminogen activator (u-PA) and their inhibition by plasminogen activator inhibitor (PAI-1) is depicted. PLG plasminogen

Contact between healthy vascular ECs and blood does not lead to adherence of platelets or clot formation, and this is not a passive phenomenon. Platelets circulate through the vascular tree in a resting state until they are needed. Therefore, ECs provide an anticoagulant and antithrombogenic boundary layer and produce actively substances that directly regulate platelet activity (Fig. 1).

One of these products that is continuously synthesized by ECs and that inhibits platelet adhesion and aggregation is nitric oxide (NO) (Radomski et al. 1987). This gas is generated from L-arginine by the endothelial isoform of NO-synthase (eNOS) in its membrane-bound configuration (Ghosh and Salerno 2003; Palmer et al. 1988). NO is membrane permeable and can diffuse into nearby platelets, where it activates guanylate cyclase (GC), which in turn converts guanosine triphosphate (GTP) to cyclic guanosine monophosphate (cGMP). The resulting increase in cGMP prevents the release of stored intracellular calcium (Ca^2+^) for platelet activation and aggregation. The production of NO by platelets themselves serves as an autocrine regulator of platelet adhesion and aggregation (Radomski et al. 1990). Furthermore, NO is also a potent vasodilator and diffuses to the vascular smooth muscle cells, where it activates GC, which leads to cGMP-mediated vasodilatation and subsequent relaxation (Furchgott and Zawadzki 1980; Vallance et al. 1989). Shear stress is a key activator of eNOS in normal physiology. In addition, the enzyme may be activated by signaling molecules, such as bradykinin, adenosine, vascular endothelial growth factor (in response to hypoxia), and serotonin (released during platelet aggregation).

Prostacyclin (prostaglandin I_2_/PGI_2_) is another effective platelet-inhibitory metabolite and vasodilator that acts independently of NO (Moncada et al. 1976, 1977). PGI_2_ is generated of arachidonic acid by the action of the cyclooxygenase (COX-1 and COX-2) and released by ECs (Wu and Liou 2005). On the platelet surface, PGI_2_ works as physiological antagonist to TxA_2_ and binds to the PGI_2_ receptor, a G protein-coupled receptor, which in turn activates intracellular adenylate cyclase (AC). Consequently, this increases the intracellular level of cyclic adenosine monophosphate (cAMP), which ultimately leads to activation of protein kinase A (PKA) and direct inhibition of Ca^2+^ mobilization and platelet granule release (Braune et al. 2020). These events limit the ability of platelets to respond to procoagulant stimuli, thereby preventing interactions with the intact vessel wall. In smooth muscle cells, PKA promotes the phosphorylation of the myosin light chain kinase, which inhibits it and leads to smooth muscle relaxation and vasodilation (Fetalvero et al. 2007). Although NO and PGI_2_ have different mechanisms of action, they synergize with each other to inhibit platelet activation and aggregation (Radomski, Palmer and Moncada 1990).

The purinergic nucleotides adenosine triphosphate (ATP), adenosine diphosphate (ADP), and adenosine monophosphate (AMP) are constitutively released from ECs at low rates, which increase at sites of vascular injury or stress. Soluble ADP released from activated platelet granules is a potent mediator for recruiting and amplifying platelet aggregation during the formation of a hemostatic plug. To regulate the activity of this agonist, vascular ECs express ectonucleoside triphosphate diphosphohydrolase-1 (E-NTPDase1/CD39), a membrane-bound enzyme that converts ATP and ADP into adenosine (Deaglio and Robson 2011; Marcus et al. 1991). In this way, ECs limit the prothrombotic signals and exert a potent, protective vascular effect.

### Heparin-antithrombin III system

A strong stimulator of platelets is thrombin (factor II), a serine protease, which is the final effector of the coagulation system and is locally produced (Mann et al. 2003). Once the coagulation has started to generate thrombin, it becomes highly amplified. Therefore, it is important that the endothelial surface provides proteins, which inhibit thrombin and counteract the progress of coagulation. Such a potent inhibitor of thrombin is the liver-derived plasma protein antithrombin III (ATIII) (Anastasiou et al. 2012). ATIII forms a complex with thrombin and other serine proteases, such as clotting factors IXa, Xa, Xia, and XIIa, and regulates them by preventing the active site of these proteases from coming into contact with their substrates. The activity of ATIII is markedly promoted by heparan sulfates (Ofosu et al. 1984). ATIII binds to specific heparan sulfates of proteoglycans in the glycocalyx that covers the endothelial surface (Bauer & Rosenberg 1991). In humans, the importance of this system has been highlighted by the occurrence of thrombotic diseases in patients with ATIII deficiency (Towne et al. 1981).

### Tissue factor pathway inhibition

Tissue factor (TF, factor III), a transmembrane protein, primarily expressed in extravascular cells and platelets but is also present in subendothelial tissue. Its role in the clotting process is the initiation of thrombin formation from prothrombin. To prevent coagulation at the top of the cascade, healthy ECs express tissue factor pathway inhibitor (TFPI), a serine protease, which limits the action of TF and inhibits excessive TF-mediated activation of coagulation factors VII and X (Girard and Broze 1993).

### Thrombin receptor thrombomodulin and the protein C/S system

To mitigate the procoagulant properties of thrombin, ECs additionally synthesize constitutively thrombomodulin (TM), a membrane-bound thrombin receptor, which directly decrease the levels of circulating thrombin (Hofsteenge et al. 1986). Upon binding to TM, thrombin undergoes a conformation change, resulting in enhanced affinity for protein C, which acts as an anticoagulant. Activated protein C forms a complex with protein S and inactivates the clotting factors Va and VIIIa (Stern et al. 1986). Thrombin bound to TM has reduced ability to convert fibrinogen to fibrin and to promote platelet aggregation (Adams and Huntington 2006). TM serves as a cofactor in the thrombin-induced activation of protein C anticoagulation pathway. Deficiency of protein C in humans has been associated with thrombotic diseases (Griffin et al. 1981). Activated protein C binds further tissue plasminogen activator inhibitor-1 (PAI-1), which was synthesized and released by ECs and plays an important role in determining the overall rate and extent of fibrinolytic process (Loskutoff and Edgington 1981).

### Fibrinolytic properties of the endothelium

ECs provide a mechanism, which counteracts the accumulation of fibrin, the result of coagulation by producing and releasing continuously tissue-type plasminogen activator (t-PA) and urokinase-type plasminogen activator (u-PA). t-PA and u-PA are serine proteases, which activates fibrinolysis by cleaving the liver-derived plasminogen (PLG) and converting it into plasmin (Loskutoff and Edgington 1977). The broadly acting protease plasmin in turn degrades cross-linked fibrin into fibrin degradation products. In this way, t-PA monitors the patency of blood vessels and removes through fibrinolysis hidden deposition of fibrin in the vessels. Modulation of t-PA release by stress, venous occlusion, thrombin, histamine, and cytokines is thought to influence directly the rate of fibrin dissolution. Furthermore, t-PA becomes acutely released from storage organelles after exposure of ECs to vasoactive agents or thrombin. The rate of fibrinolysis is limited by the steady-state availability of t-PA during fibrin-polymerization. The efficacy of t-PA within the plug is much higher than that of t-PA molecules added from outside to existing plug (Medcalf 2007). In general, the repertoire of all these coagulation inhibitors expressed in ECs can vary according to different organs and even within the vasculature of an organ (Aird 2015).

## Endothelial injury

### Hemostasis

At sites of vascular injury, the endothelium shifts from an anticoagulant to a procoagulant/prothrombotic phenotype to prevent excessive blood loss. Vasoconstriction is an important initial response when the vessel is damaged. It is caused by direct exposition of smooth muscle cells to locally generated vasoactive agents, such as bradykinin, histamine, vasopressin, or thrombin, and by bypassing the vasodilatory action of ECs (Durand and Gutterman 2013). This leads to reduction of the vessel diameter and a slowdown in blood flow, which is the hemodynamic basis for subsequent processes. Coagulation begins almost instantly, a process resulting in hemostasis, the cessation of blood loss, followed by repair. In hemostasis, two components are involved, platelets and the coagulation system. Disruption of the endothelial continuity leads to exposure of collagen fibers and other subendothelial matrix proteins (Ruggeri 2002). Circulating platelets rapidly adhere to these structures and start the hemostatic process. Platelets immediately aggregate to form thrombi. Therefore, platelets have a wide array of surface receptors and adhesion molecules and contain numerous granules (Saboor et al. 2013; Sharda and Flaumenhaft 2018). Additional coagulation (clotting) factors respond in a cascade to form fibrin strands, which strengthen the platelet plug (Macfarlane 1964).

### Platelet adhesion

**Fig. 2:**
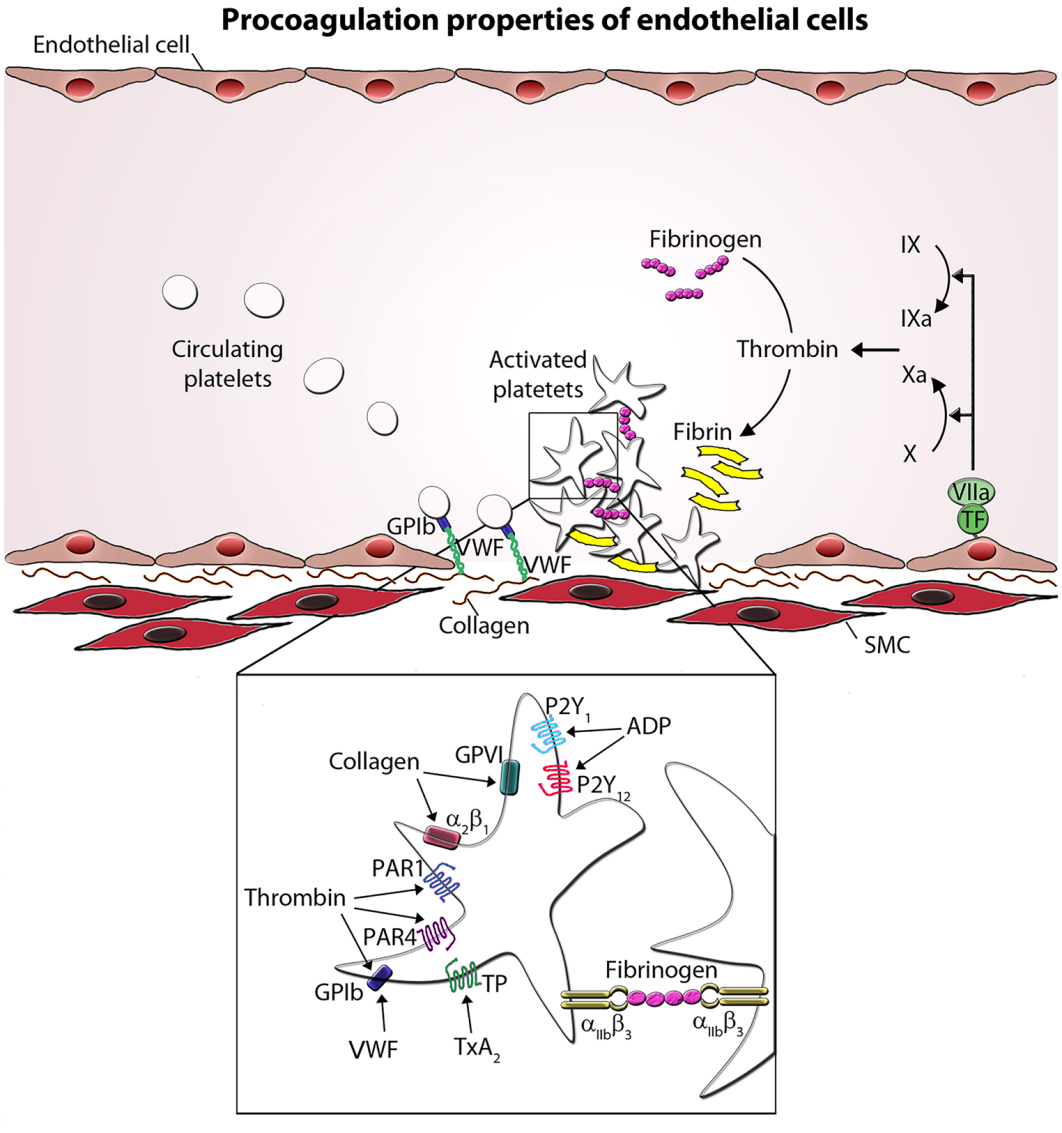
Procoagulation properties of endothelial cells. At sites of vascular injury, Von Willebrand factor (VWF) binds to the exposed subendothelial collagen. VWF then captures platelets from the circulation via the interaction of the VWF with GPIb on the platelet. The binding of GPVI with collagen leads to further platelet activation. This results in the integrin-mediated adhesion of platelets and cellular activation releasing mediators. Selected platelet agonists and their receptors are indicated. Thrombin binds to GPIb and activates protease-activated receptor 1 and 4 (PAR1 and PAR4, respectively), ADP activates P2Y_1_ and P2Y_12_ receptor, and thromboxane A_2_ (TxA_2_) activates the thromboxane receptor (TP). Agonist stimulation results in integrin α_IIb_β_3_ activation and the transition to an active conformation, which binds fibrinogen and mediates platelet aggregate formation. SMC smooth muscle cell

Platelet adhesion to the extracellular matrix arranges a first, thin cover of the defect ECs. In this process, the von Willebrand factor (VWF) is essential (Sakariassen et al. 1979). VWF is a multimeric glycoprotein and is synthesized by ECs and stored in characteristic endothelial ultrastructural features, the Weibel-Palade bodies (Weibel and Palade 1964). VWF is also synthesized by megakaryocytes and localized in α-granules of platelets (Wagner and Marder 1984). Injured ECs release VWF by vasoactive agents, such as bradykinin, histamine, vasopressin, or thrombin, which enhance the cytoplasmic Ca^2+^ level and activate protein kinase C (Birch et al. 1992). During this release process, Weibel-Palade bodies fuse with the plasma membrane and release VWF. Under the conditions of high shear stress, plasma VWF binds with its A3-domain to exposed subendothelial collagen and participates in interaction between platelets and ECs. This process induces uncoiling of VWF, and VWF exposes its A1-domain, which binds platelet VWF receptor, the glycoprotein (GP)Ib-V-IX complex. VWF serves as a bridge between subendothelial collagen and the platelet via GPIbα (subunit of the GPIb-V-IX) complex) at the platelet surface (Lankhof et al. 1996) (Fig. 2).

Therefore, it is not surprising that defects or dysfunction of the GPIb-V-IX complex are associated with a bleeding disorder, named Bernard-Soulier syndrome, which is characterized by thrombocytopenia and giant platelets. As a consequence, platelets are unable to adhere and a bleeding phenotype results (Zieger et al. 2009). The von Willebrand disease is caused by a quantitative a quantitative (reduction of VWF antigen) or qualitative (reduction of VWF activity) defect of VWF and is also characterized by bleeding symptoms (Goodeve and James 1993).

Interestingly, the interaction between subendothelial collagen, VWF, and platelets via the GPIb-V-IX complex is transient and does not lead to a stable adhesion; however, it facilitates efficient recruitment of platelets from the circulation to the damaged area (Savage et al. 1996). The localization of platelets close to the extracellular matrix allows now direct collagen interaction with the platelet membrane receptor GPVI (Nieswandt and Watson 2003). Binding of GPVI to collagen triggers the activation of the second platelet collagen receptor α_2_β_1_ (GPIa/IIa), which is important for firm adhesion of platelets to the collagen surface (Kainoh et al. 1992) and of the platelet fibrinogen receptor integrin α_IIb_β_3_, which further strengthens adhesion (Goto et al. 2002). When platelets adhere to the subendothelial matrix, they change their shape from discoid to spherical (Ehrman et al. 1978). The platelet cytoplasm expands, and the cytoskeleton rearranges to develop fingerlike protrusions, filopodia and lamellipodia. The adhesion receptors are redistributed in lipid rafts and concentrate to the terminal parts of filopodia. These changes in shape are essential to make platelets more adherent to the injured area and to other platelets, thus increasing the surface area of platelets protecting the injured endothelium and preventing further blood loss.

### Platelet activation

Interaction of collagen with platelets via GPVI and α_2_β_1_ induces intracellular signaling and potentiates platelet leading to α- and δ-granule secretion into the blood plasma. Platelet α-granules contain adhesive glycoproteins, such as fibrinogen, VWF, coagulation factors, P-selectin, angiogenetic factors, mitogenic factors, fibrinolytic inhibitors, and immunoglobulins. δ-granules are the storage pool of a variety hemostatically active nonprotein molecules, which are released during platelet activation. Those include catecholamines like serotonin and histamine, ADP, ATP, and Ca^2+^. Secreted agonists in turn activate surrounding platelets and trigger additional degranulation in terms of a positive feedback loop during platelet activation (Flaumenhaft 2003; Ruggeri 2002).

ADP released from damaged endothelial cells and δ-granules from activated platelets plays a central role in regulating platelet function. Via their platelet purinergic receptors, P2Y_1_ and P2Y_12_, ADP causes platelet shape change, aggregation, and generation of thromboxane A_2_ (TxA_2_), another platelet agonist (Packham & Mustard 2005).

Analogous to ADP, TxA_2_ can also (auto-)activates platelets. Upon platelet activation, TxA_2_ is synthesized from arachidonic acid, which is liberated from the plasma membrane through conversion by cyclooxygenase-1 (COX-1). TxA_2_ interacts with the thromboxane A_2_ receptor (TP) (FitzGerald 1991).

Another strong stimulator of platelets is thrombin, which is the final product of the coagulation system and responsible for converting fibrinogen into fibrin to stabilize the platelet plugs (Mann, Butenas and Brummel 2003). Thrombin interacts with protease-activated receptor 1 and 4 (PAR1 and PAR4), which enhance the release of VWF. This results in exposure of adhesion molecules at the platelet surface (ICAM-1, VCAM-1, E-selectin, P-selectin) and production of plasminogen activator inhibitor type-1 (PAI-1) (Leger et al. 2006). ECs express PAI-1, which functions as principal inhibitor of t-PA and u-PA activity and prevents fibrinolysis (Sprengers and Kluft 1987). Elevated levels of circulating PAI-1 are a risk factor for thrombosis and atherosclerosis (Vaughan 2005).

Additionally, ECs and platelets synthesize platelet-activating factor (PAF), a phospholipid mediator that promotes the adhesion of platelets to the ECs and their activation and aggregation (Agarwal et al. 1994).

During activation, platelets secrete serotonin (5-hydoxytryptamine, 5-HT) from their δ-granules, which is considered a weak platelet agonist, but with the ability to enhance the aggregation-response of platelets to other agonists, like ADP (Vanags et al. 1992).

These described soluble agonists trigger platelet activation through their G protein coupled receptors, resulting in increased Ca^2+^ concentrations in the platelets' cytosol which in turn activate specific signaling pathways promoting plug formation (Offermanns 2006; Varga-Szabo et al. 2009).

Platelet activation further results in redistribution of phospholipids to their outer membrane, for example, phosphatidylserine, which is essential for platelet adhesion and thrombus formation, since it promotes assembly of prothrombinase complexes on the platelet surface (Heemskerk et al. 1997).

### Platelet aggregation

Aggregation of platelets begins shortly after activation and occurs as a result of the conformational change of the fibrinogen receptor integrin α_IIb_β_3_ (GPIIb/IIIa) at the platelet surface (Joo 2012; Nieswandt et al. 2009). In resting platelets, α_IIb_β_3_ exists in an inactive conformation, unable to bind to its circulating plasma ligands, fibrinogen, and VWF. During activation, the extracellular domain of α_IIb_β_3_ transits from its resting to an active form by phosphorylation of its cytoplasmic domain, a process called “inside-out” signaling, resulting in increased affinity for its ligands. Furthermore, the surface density of active α_IIb_β_3_ increases during platelet activation. Bound fibrinogen or VWF to functional α_IIb_β_3_ cross-links platelets and is essential for triggering then “outside-in signaling” and subsequent thrombus formation and stabilization. The importance of integrin α_IIb_β_3_ becomes apparent in a severe platelet disorder named Glanzmann thrombasthenia that is characterized by lack of platelet aggregation. The molecular basis is linked to quantitative and/or qualitative abnormalities of α_IIb_β_3_ (Sandrock-Lang et al. 2015).

Once a platelet plug has been formed, activation of the coagulation cascade is necessary to stabilize this plug by producing a fibrin mesh. Coagulation requires the sequential activation of blood-based serine proteases and their cofactors (blood clotting factors).

### Coagulation cascade

Activation of the coagulation cascade results in the formation of fibrin from the plasma protein fibrinogen (Macfarlane 1964). The major initiator of this clotting process is TF. In the intact vessel wall, TF is hidden and is exposed to blood in response to injury. Platelets and coagulation factors leak into the subendothelial compartment, where membrane-bound TF can directly bind to coagulation factor VII. The TF-VIIa complex converts circulating factor IX and X into active enzymes, factors IXa and Xa (Fig. 2). Factor IXa further enhances the activation of factor X. Factor Xa subsequently catalyzes the generation of thrombin (factor IIa) from the zymogen prothrombin. Thrombin is the key enzyme since it cleaves fibrinogen into fibrin. Fibrin polymerizes into a fibrin network, which forms the blood clot with the aggregating platelets. Furthermore, thrombin is responsible for the conversion of factors V, VIII, XI, and XIII into their active form. VWF plays not only a major role in initial platelet recruitment but also in formation of the fibrin clot by associating with VIII and preventing its rapid clearance from the circulation (Tuddenham et al. 1982). When healing progresses, the thrombus can be degraded again by proteases, especially plasmin.

## Conclusion

Vascular ECs form a dynamic lining at the interface between tissue and blood. They maintain blood fluidity and protect against injury of the vessel wall through delicate regulation of platelet activity, blood coagulation, and thrombolysis. Their effect on coagulation is the resultant or balance of multiple mechanisms, both anticoagulant and procoagulant. Loss of normal endothelial function or dysfunction of platelet may result in severe bleeding symptoms in humans (Sandrock-Lang et al. 2016).
